# Whole-body tumor segmentation from FDG-PET/CT: Leveraging a segmentation prior from tissue-wise projections

**DOI:** 10.1016/j.heliyon.2024.e41038

**Published:** 2024-12-10

**Authors:** Sambit Tarai, Elin Lundström, Nouman Ahmad, Robin Strand, Håkan Ahlström, Joel Kullberg

**Affiliations:** a Radiology, Department of Surgical Sciences, Uppsala University, Uppsala, SE-75185, Sweden; b Department of Information Technology, Uppsala University, Uppsala, SE-75237, Sweden; c Antaros Medical AB, Mölndal, SE-43153, Sweden

**Keywords:** Whole-body tumor segmentation, Multi-channel multi-angled PET/CT projections, Backprojection, Segmentation prior

## Abstract

**Background**:

Accurate tumor detection and quantification are important for optimized therapy planning and evaluation. Total tumor burden is also an appealing biomarker for clinical trials. Manual examination and annotation of oncologic PET/CT is labor-intensive and demands a high level of expertise. One significant challenge is the risk for human error, leading to potential omission of especially small tumors and tumors with low FDG uptake.

**Purpose**: In this study, we introduced an automated framework with segmentation prior, from a tissue-wise multi-channel multi-angled based approach, to enhance tumor segmentation in whole-body FDG-PET/CT.

**Method**: The proposed framework utilized a segmentation prior generated from tumor segmentations in tissue-wise multi-channel projections of the standardized uptake value (SUV) from PET. Projections were created from various angles and the tissues were identified based on their CT Hounsfield values. The resulting segmentation masks were subsequently backprojected into a unified 3D volume for creation of the segmentation prior. Finally, the segmentation prior was provided as an additional input channel along with the CT and SUV images to three variants of 3D segmentation networks (3D UNet, dynUNet, nnUNet) to enhance the overall tumor segmentation performance. All the methods were independently evaluated using 5-fold cross-validation on the autoPET dataset and subsequently tested on the U-CAN dataset.

**Results**:

Combining the segmentation prior with the original SUV and CT images improved overall tumor segmentation performance significantly compared to a baseline network. The increase in Dice coefficient for lymphoma, lung cancer, and melanoma across different segmentation networks were: 3D UNet ($0.04^{⁎}$, $0.02^{⁎}$, $0.11^{⁎}$), dynUNet ($0.05^{⁎}$, $0.04^{⁎}$, $0.08^{⁎}$), and nnUNet ($0.02^{⁎}$, $0.00^{ns}$, $0.03^{⁎}$), respectively; *, p-value < 0.05; ns, non-significance.

**Conclusion**: The increased segmentation accuracy could be attributed to the segmentation prior generated from tissue-wise SUV projections, revealing information from various tissues that was useful for segmentation of tumors. The results from this study highlight the potential of the proposed method as a valuable future tool for time-efficient quantification of tumor burden in oncologic FDG-PET/CT.

## 1 Introduction

According to the World Health Organization (WHO), cancer stands as one of the leading causes of death worldwide, surpassing all other health related disorders [1]. Each year, the number of individuals diagnosed with cancer continues to rise, emphasizing its escalating prevalence. While detecting the presence of cancer may not be overly challenging, accurate quantification of tumors at an early stage remains critical, especially identification of small and low contrast metastases emerging in different parts of the body. In clinical practice, diagnostic assessments, staging, and monitoring of certain cancer forms can be performed non-invasively using positron emission tomography combined with computed tomography (PET/CT) after injecting 18F-fluorodeoxyglucose (FDG) [2]. FDG is widely used in routine oncologic PET due to its sensitivity to the high glucose metabolism of malignant tumors.

Traditionally, tumor segmentation has relied solely on manual delineation of FDG-PET/CT images by radiologists. As a result, it has become labor-intensive, time-consuming, and susceptible to human errors [3]. Furthermore, there is a risk of the radiologists overlooking small lesions and lesions with low FDG uptake, which can have serious consequences as they can proliferate over time and spread. Therefore, early and precise lesion detection becomes vital for non-invasive tumor tracking as a step in streamlining the treatment planning and potentially improving patient outcome. Additionally, this is important for estimating the total metabolic tumor volume (TMTV), quantifying the total number of lesions and their locations in the body, detecting the presence of new lesions in follow-up scans, and assessing lesion-specific changes post-treatment. These are important prognostic factors in risk assessment, therapy optimization and evaluation [4]. They are also appealing biomarkers for clinical trials.

Several convolutional neural network (CNN) based architectures have been developed for image segmentation, with UNet [5] being most widely used. Expanding on UNet, Zhou et al. introduced nested and dense skip connections in their network called UNet++ [6] [7], aiming at reducing the semantic gap between the encoder and decoder for improved segmentation results. Recently, Isensee et al. developed the nnUNet [8] architecture, featuring a self-adapting framework for configuring various segmentation components automatically. DynUNet is another segmentation network provided by MONAI [9], an open source framework. It builds upon the foundations of nnUNet and delivers exceptional performance with ease of implementation.

Recent studies have made significant advancements in automated PET/CT tumor segmentation, including the development of deep transfer learning approaches and ISA-Net, which have shown effectiveness in quantifying molecular tumor burden quantification, risk stratification, and treatment response evaluation [10] [11]. The HECKTOR challenge at MICCAI 2020 further highlighted the progress in segmenting Gross Tumor Volume (GTV) in head and neck cancer using FDG-PET/CT, where top methods outperformed human inter-observer agreement [12]. Similarly, the autoPET challenge at MICCAI 2022 confirmed the feasibility of accurate automated segmentation of metabolically active tumors in whole-body PET/CT, with success largely dependent on data quality and quantity [13].

Despite significant advancements in the field of tumor segmentation from medical imaging [13] [14] [12] [15] [16] [17], some challenges persist including diverse tumor characteristics, anatomical misalignment between PET and CT, limited inter-operator agreement between radiologists during delineation and uncertainty in the annotation boundary. Among all, the most crucial challenge faced by many networks is the accurate segmentation of small and low FDG uptake lesions. Therefore, the main goal of this work was to develop an automated framework that effectively segments challenging lesions overlooked by conventional baseline networks, surpassing current state-of-the-art methods. The developed solution has the potential to assist radiologists by reducing their workload and minimizing the risk of overlooking critical information during diagnostics. Additionally, it can support longitudinal monitoring of cancer patients, contributing to improved patient care.

Segmentation prior-based tumor segmentation in whole-body PET/CT and PET/MRI datasets has previously been explored by our reseach group [18]. This includes segmentation of tumors from multiple 2D SUV maximum intensity projections (MIPs) in order to generate a segmentation prior, thereafter used as an independent input channel for tumor segmentation in 3D. In this case, the segmentation prior consists of a single channel, corresponding to the SUV MIP from all tissues, projected at multiple angle (i.e. single-channel multi-angle approach). In segmentation prior-based methods, the effectiveness of the 3D tumor segmentation framework depends on the quality of the segmentation prior. Improving the reconstruction of such segmentation priors can result in enhanced tumor segmentation performance. By separating voxels from different tissues, tissue-wise SUV MIPs at multiple angles (i.e. a tissue-wise multi-channel multi-angle approach) can be obtained for increased information content in the projections, which potentially can assist in the tumor segmentation. Therefore, building upon previous work, our primary goal is to utilize a tissue-wise multi-channel multi-angle PET/CT projection-based approach [19] to improve the quality of the segmentation prior, aiming for state-of-the-art tumor segmentation results in whole-body FDG-PET/CT.

Previous methods have relied on training different variants of the UNet using extensive PET/CT and other datasets [20], [21], [22], [23], [24], [25], [26], [27], [28]. However, such methods may be clinically less relevant as different cancer types can exhibit heterogeneous imaging characteristics, necessitating disease-specific training for a more realistic approach. Hence, one of the secondary aims of this paper was to conduct disease-wise training, wherein independent neural networks were trained for different cancer types.

The main objectives of the paper can be summarized as follows:

1. Developing an automated tumor segmentation framework, using three different 3D segmentation networks, to evaluate the advantages of the proposed method with various architectures.
2. Applying a tissue-wise multi-channel PET/CT projection-based approach to enhance the quality of the segmentation prior.
3. Investigating the benefits of disease-specific training versus general training (the latter including all cancer types).
4. Comparing different models (baseline and proposed) through voxel-wise and lesion-wise analysis of segmentation metrics.
5. Independently testing various approaches (baseline, prior_1, prior_2) on an internal test set to assess their generalizability.

## 2 Methodology

### 2.1 Dataset

This study utilizes FDG-PET/CT images from the autoPET cohort [29] for the purpose of comprehensive tumor segmentation analysis and validation of the performance of the proposed method against the baseline method. It also uses an internal test set from the U-CAN cohort [30] to evaluate the generalizability of the developed models. Table 1 provides an overview of the key features of the datasets utilized in the study. Ethical approval was obtained from the Swedish Ethical Review Authority to conduct retrospective image analysis on both datasets.

**Table 1:** Summary of FDG-PET/CT datasets.

Parameters	autoPET	U-CAN
Medical imaging	FDG-PET/CT	FDG-PET/CT
Examinations	501	68
Cancer types	Lymphoma, Lung cancer, Melanoma	Diffuse large B cell lymphoma
Sex (Male/Female)	(290/209)	(51/37)
Avg. total metabolic tumor volume	220 ml	107 ml
Number of cites	single-cite	multi-cite

CT scanner	Siemens Biograph mCT	-
CT mAs	200 mAs	-
CT Tube Voltage	120 kV	-
CT Contrast Agent	Ultravist 370	-

PET Radioactivity	314.7 MBq	-
PET Acquisition Time per Bed Position	2 minutes	-

#### 2.1.1 autoPET cohort

The autoPET dataset originated from a medical center in Germany. This dataset comprises three different cancer types: lymphoma (144 scans), lung cancer (167 scans), and melanoma (188 scans), as well as a negative control group (513 scans). The voxel size in each image is (2.04 x 2.04 x 3.00) mm^3^. All PET/CT images and their manual annotations are provided as 3D volumes, typically ranging from the head to the mid-thigh level, and in some cases, the entire body as per clinical relevance. The manual annotations were conducted by two expert radiologists with ten and five years of experience. The dataset is publicly available at TCIA (The Cancer Imaging Archive) [31].

#### 2.1.2 U-CAN cohort

A subset of the U-CAN dataset, consisting of 65 whole-body FDG-PET/CT images of Diffuse large B cell lymphoma (DLBCL) patients was used as a test set. The voxel size in each of the image is (2.04 x 2.04 x 3.00) mm^3^. Manual annotations were performed by a medical student under the supervision of a radiologist with 5 years of experience. All annotations were approved by the radiologist. The dataset belongs to the U-CAN consortium and is not publicly accessible.

### 2.2 Data pre-processing

The PET data underwent a standardization process by converting the voxel intensities to standardized uptake value (SUV) normalized by body weight. All CT and their corresponding SUV images were resampled to a common imaging resolution, ensuring uniform spacing. The voxel intensities were clipped between [-100, 250] for CT and [0, 15] for SUV, and thereafter normalized between [0, 1].

### 2.3 Overview of the proposed tumor segmentation framework

**Figure 1:**
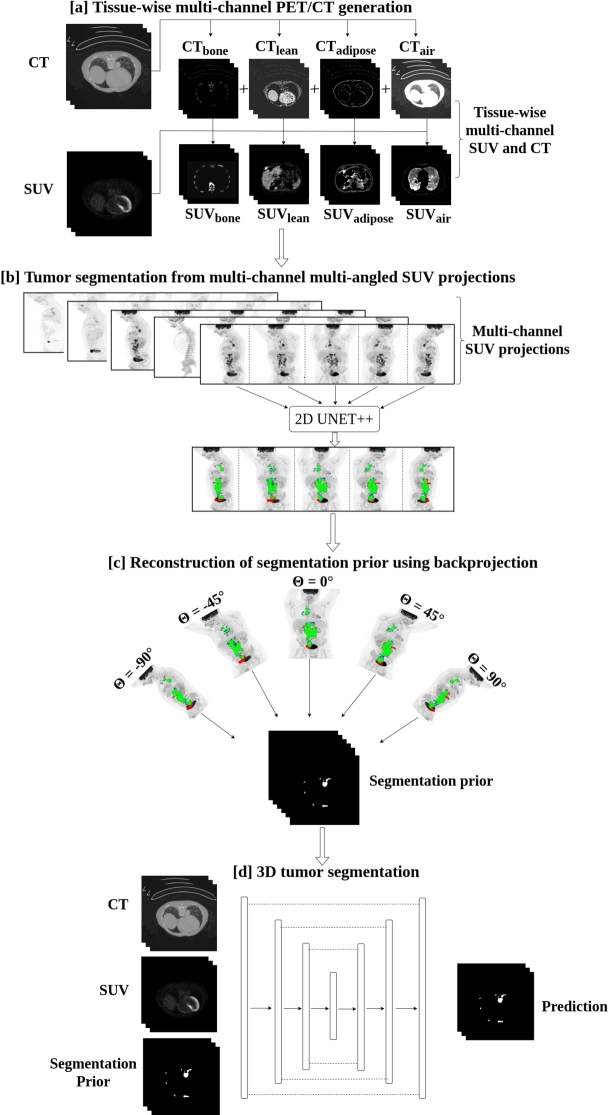
Overview of the proposed framework for automated tumor segmentation from whole-body FDG-PET/CT: (a) Generation of tissue-wise multi-channel inputs from SUV and CT images, (b) Segmentation of tumors in 2D projections using multi-channel multi-angled SUV MIPs, (c) Generation of segmentation prior, (d) 3D Tumor segmentation.

The overall workflow for the automated tumor segmentation comprised four steps: (a) Generation of tissue-wise multi-channel inputs from SUV and CT images, (b) Segmentation of tumors in 2D projections using multi-channel multi-angled SUV MIPs, (c) Generation of segmentation prior, and (d) 3D Tumor segmentation. These steps are illustrated in Fig. 1 and described into detail in subsections [a] - [d] below.

#### 2.3.1 Tissue-wise multi-channel PET/CT generation

In the first step, all original CT images were categorized into different tissues: bone, lean tissue, adipose tissue and air, according to eqs. (1) (2) (3) (4) [32] [33]. This process resulted in the creation of multi-channel binary CT masks, hereafter referred to as “bone”, “lean”, “adipose”, and “air”.

(1) $$CT_{i}(bone)=\left\{ \begin{array}{cc} 1 & \text{if }i\geq 200\\ 0 & \text{elsewhere} \end{array}\right.$$

(2) $$CT_{i}(lean)=\left\{ \begin{array}{cc} 1 & \text{if }i\in [-29,150]\\ 0 & \text{elsewhere} \end{array}\right.$$

(3) $$CT_{i}(adipose)=\left\{ \begin{array}{cc} 1 & \text{if }i\in [-190,-30]\\ 0 & \text{elsewhere} \end{array}\right.$$

(4) $$CT_{i}(air)=\left\{ \begin{array}{cc} 1 & \text{if }i<-190\\ 0 & \text{elsewhere} \end{array}\right.$$

where i represents the voxel intensity. These CT masks were utilized to derive tissue-specific multi-channel CT and SUV images by voxel-wise multiplication between the masks and the corresponding ${\text{CT}}_{\text{orig}}$ and ${\text{SUV}}_{\text{orig}}$ images, respectively. This approach effectively isolated the respective tissues. As a result, several multi-channel inputs were obtained, including the ${\text{CT}}_{\text{orig}}$, ${\text{SUV}}_{\text{orig}}$, ${\text{CT}}_{\text{bone}}$, ${\text{SUV}}_{\text{bone}}$, ${\text{CT}}_{\text{lean}}$, ${\text{SUV}}_{\text{lean}}$, ${\text{CT}}_{\text{adipose}}$, ${\text{SUV}}_{\text{adipose}}$, ${\text{CT}}_{\text{air}}$, and ${\text{SUV}}_{\text{air}}$ (as shown in Fig. 1 [a]). To ensure consistency and comparability among different channels, all the multi-channel inputs, except for the ${\text{CT}}_{\text{orig}}$ and ${\text{SUV}}_{\text{orig}}$, were subsequently normalized within the range of [0, 1]. The data pre-processing step mentioned in Section 2.2 was applied to ${\text{CT}}_{\text{orig}}$ and ${\text{SUV}}_{\text{orig}}$.

The different ranges of CT Hounsfield units (HUs), shown in eqs. (1) (2) (3) (4), have previously been used in medical imaging to visualize the specific tissues of interest [32] [33]. For example, a bone window can be used to enhance contrast in bone tissue for easier identification of lesions and abnormalities within the skeletal system and a lean tissue window can visualize soft tissue lesions present in muscles, organs, and vessels, aiding in detection and characterization of such abnormalities.

#### 2.3.2 Tumor segmentation from multi-channel multi-angled SUV projections

MIPs were generated from the ${\text{SUV}}_{\text{orig}}$ volume as well as from the different SUV channels, with respect to the axial direction within the range of angles [-90°, 90°) and with consecutive projections created at 10° intervals [34]. A total of 18 different predetermined projections were generated for each patient, resulting in 9018 different projections (from 501 scans) obtained from the autoPET cohort (according to [18]). Each projection contained 5 different channels ($SUV_{org}^{MIP}$, $SUV_{bone}^{MIP}$, $SUV_{lean}^{MIP}$, $SUV_{adipose}^{MIP}$, $SUV_{air}^{MIP}$). The combination of all multi-channel projections with multiple angles is referred to as multi-channel multi-angled projections. Additionally, the corresponding ground truth segmentation labels to the MIPs were generated following a similar multi-angle approach, which resulted in 18 different ground truth masks per scan. The ground truth labels were 2D binary masks where the foreground pixels represented tumors in each of the multi-channel projections while background pixels corresponded to non-tumor regions.

A 2D UNet++ [6] [7] was employed for segmenting the tumor regions from these multi-channel multi-angled projections (as shown in Fig. 1 [b]). The network uses the 5-channel projection image as input and is optimized to segment any existing lesion. Due to variations in the field of view, the image size of the projections differs significantly among patients within this cohort. Because of this reason, a batch size of 1 was utilized during training to accommodate for varying sizes of the projections. To optimize the training process, a combination of Dice and Focal loss functions was used [9] [35] [36] [37]. The Adam optimizer was chosen, with a learning rate of 1e-4. Additionally, a weight decay of 1e-5 was employed to regulate the weight magnitudes and mitigate potential overfitting. To introduce regularization, a dropout rate of 0.20 was used.

Potentially, most of the tumor-related information is available in the $SUV_{orig}^{MIP}$ channel itself. Therefore, we investigated whether inclusion of the additional 4 channels (bone, lean, adipose, air) were of use during the tumor segmentation process from the 2D projections. In order to analyze this, we trained two different networks (see Table 2), one which only uses the $SUV_{orig}^{MIP}$ as input and a second that uses the 5 channel image as input ($SUV_{orig}^{MIP}$, $SUV_{bone}^{MIP}$, $SUV_{lean}^{MIP}$, $SUV_{adipose}^{MIP}$, $SUV_{air}^{MIP}$).

**Table 2:** Results of 2D tumor segmentation using multi-channel multi-angled projections.

Disease	Inputs					Dice
	MIP	Bone	Lean	Adipose	Air	
Lymphoma	✓					0.6587
Lung Cancer	✓					0.7356
Melanoma	✓					0.5824

Lymphoma	✓	✓	✓	✓	✓	0.6869
Lung Cancer	✓	✓	✓	✓	✓	0.7667
Melanoma	✓	✓	✓	✓	✓	0.6148

To optimize the results, two distinct network combinations were trained: disease-specific training and general training. In the disease-specific training approach, three separate networks were trained on the three cancer types, allowing them to learn disease-specific features. In the general training approach, a single network was trained on all disease types, enabling it to capture more general tumor-related features. The rationale behind this approach was to benefit from the strengths of both network configurations. By training disease-specific networks, each model can specialize in learning features relevant to its respective cancer type, potentially enhancing its ability to detect specific characteristics unique to that disease. Conversely, the general training approach benefits from a larger and more diverse training data (from all three cancer types), which enables the network to learn more general tumor-related features that may be applicable across different cancer types. Finally, an ensemble of the above two network combinations were employed for the final prediction. Here, the objective was to maximize the detection of independent lesions (present in the projection), while effectively filtering out apparent false positives.

#### 2.3.3 Reconstruction of segmentation prior using backprojection

After segmenting the lesions from the 2D projections, a backprojection algorithm [38] [39] was employed to reconstruct a volumetric representation of the lesion locations, called the “segmentation prior” (as shown in Fig. 1 [c]). This approach involved combining the 2D segmentation predictions obtained from 18 predetermined 2D projections for a given patient. The information from different angles was aligned and fused to trace the foreground pixels, corresponding to the predicted lesions, back to their original location in 3D [40] [41]. This process was performed for all 18 projections, resulting in 18 distinct 3D volumes, each associated with a specific 2D segmentation mask. Subsequently, all 18 volumes were combined into a single 3D volume by summing them together followed by multiplying the resultant with the corresponding SUV volume. This aggregation enhanced the contrast of overlapping regions, intensifying their representation compared to the non-overlapping regions. The resulting 3D backprojected volume is known as the segmentation prior. It aimed to provide a comprehensive and enriched representation of the lesions present in the whole body, incorporating information from different tissues and multiple angles. This could facilitate the understanding of the spatial distribution and characteristics of the lesions in a 3D context, surpassing the limitations of the initial 2D segmentations. The segmentation prior is essentially a 3D volume with the same image resolution and matrix size as the original PET/CT image, containing prior information about tumor characteristics such as size, shape, and location. The intensity values of the segmentation prior were normalized between the range [0, 1]. Furthermore, the bottom 5 percentile of intensity values, which primarily corresponded to noise, were removed.

During the reconstruction process, it is crucial to ensure that as many lesions as possible, ideally all of them, are highlighted in the resulting segmentation prior, thus providing a comprehensive and accurate depiction of the lesion locations. Segmenting tumors from tissue-wise multi-channel multi-angled 2D projections not only aims to enhance the visibility of the maximum number of lesions within the segmentation prior but also to minimize false positives. Two different types of segmentation priors were created. The first, referred to as “segmentation prior 1” was generated using only the $SUV_{orig}^{MIP}$ as input (further details provided in [18]). The second, referred to as “segmentation prior 2” was created using the multi-channel multi-angled 2D projections (such as $SUV_{orig}^{MIP}$, $SUV_{bone}^{MIP}$, $SUV_{lean}^{MIP}$, $SUV_{adipose}^{MIP}$, and $SUV_{air}^{MIP}$), as previously discussed in section 2.3 (b).

#### 2.3.4 3D tumor segmentation

In the final step, the goal was to perform whole-body tumor segmentation in 3D (as shown in Fig. 1 [d]). For this, three 3D UNet models were evaluated, all with the same network architecture, except for the number of input channels. The first model, referred to as 3D UNet (baseline), utilized two input channels: the CT and the SUV. The second model, named 3D UNet (prior_1), utilized three input channels: CT, SUV, and “segmentation prior 1.” The third model, named 3D UNet (prior_2), also used three input channels: CT, SUV, and “segmentation prior 2.” The evaluation aimed at studying the efficacy of different segmentation priors in improving tumor segmentation performance when compared to the baseline model. To evaluate the effectiveness of incorporating the segmentation priors, the study also included training different variants of the 3D segmentation network architecture, such as dynUNET [9] and nnUNET [8], in a benchmarking study alongside the 3D UNet model, using corresponding input channels. The standard architectures were used without any modifications. Throughout the paper, the term “baseline” referred to the 3D segmentation network with two input channels, while “prior_1” and “prior_2” referred to the 3D segmentation networks with three input channels, based on the specific segmentation priors utilized. To summarize, the study focused on evaluating three independent network architectures, 3D UNet, dynUNet, and nnUNet, on three different cancer types: lung cancer, lymphoma, and melanoma. Within each of the network architectures, three variants of the segmentation models were trained, referred to as baseline, prior_1, and prior_2. The variations in these models were limited to the number and types of input channels, allowing for a comprehensive assessment of the impact of segmentation priors.

To ensure consistency and unbiased evaluation, all models were independently assessed using five-fold cross-validation, employing the same training-validation split throughout the process. Stratification based on sex was also applied to each of the five folds to maintain the same distribution between males and females in each fold. To achieve optimal performance, disease-specific training was conducted for all the models. During the training phase, the networks dedicated to lymphoma were trained from start without any pre-training where-as for the other cancer types, the lymphoma network served as an initialization step for pre-training. All the models were trained for 300 epochs to ensure consistency except the nnUNet models, which was trained for 1000 epochs. During training, a patch size of (160, 160, 160) voxels was used, with patches extracted through a sliding window approach with an overlap of 0.25 between consecutive patches. The training process employed the Dice focal loss function [36] [37], optimized using the Adam optimizer, with a learning rate of 1e-4, weight decay of 1e-5, drop out rate of 0.20 and a batch size of 1. The experiments were conducted on a machine equipped with 32 GB of internal RAM and an Nvidia RTX 3090 Ti GPU with 24 GB of memory.

### 2.4 Evaluation metrics

The performance of different tumor segmentation models were evaluated using the following metrics, as presented in equations (5), (6), (7), (8), and (9): Dice coefficient, Hausdorff distance (HD95), Average surface distance (ASD), lesion-wise recall, and lesion-wise precision. Lesion-wise precision is the ratio of the number of independent lesions correctly detected by the network to the total number of lesions predicted by the network. Lesion-wise recall is the ratio of the number of independent lesions correctly detected by the network to the total number of lesions present in the ground truth. Lesion-wise precision and recall were estimated by extracting the total number of independent lesions using connected component analysis. Clusters of connected components were found using a 27-connected neighborhood in 3D, and only connected components with volumes greater than 0.3 ml were considered for the analysis.

If G represents the ground truth label, P represents the prediction by the network and TP, FN, FP correspond to the independent true positive, false negative, false positive lesions, then the above metrics can be defined as follows:

(5) $$\text{Dice}=\frac{2\times |A\cap B|}{\left|A|+|B\right|}$$

(6) $$\text{HD}=\max\left(\max_{p\in \text{P}}\min_{q\in \text{G}}\text{distance}(p,q),\max_{q\in \text{G}}\min_{p\in \text{P}}\text{distance}(p,q)\right)$$

(7) $$\text{ASD}=\frac{1}{N}\sum_{i=1}^{N}\left(\min_{p\in \text{P}}\text{distance}(p,q_{i})+\min_{q\in \text{G}}\text{distance}(p_{i},q)\right)$$

(8) $$\text{Recall}=\frac{\text{TP}}{\text{TP}+\text{FN}}$$

(9) $$\text{Precision}=\frac{\text{TP}}{\text{TP}+\text{FP}}$$

### 2.5 Statistics

Two-sided Wilcoxon signed-rank test was conducted to study potential differences in the performance between the three models. A p-value less than 0.05 was considered statistically significant.

## 3 Results

### 3.1 2D tumor segmentation using multi-channel multi-angled SUV projections

Table 2 presents the results from five-fold cross-validation on three cancer types using multi-channel multi-angled SUV projections. The table provides a comparison of the 2D tumor segmentation performance, using all five SUV input channels against using only the $SUV_{org}^{MIP}$ channel. Results show better performance with all five input channels as opposed to a single $SUV_{org}^{MIP}$ channel, suggesting segmentation prior 2's superiority over segmentation prior 1. Additionally, Both methods effectively segmented tumors from one or more projection angles.

### 3.2 3D tumor segmentation

Table 3 presents the results from five-fold cross-validation across all three cancer types with different methodologies (baseline, prior_1, prior_2) using the 3D UNet model. Table 4, Table 5 presents similar results with different network architectures (dynUNet and nnUNet). In general, the models based on prior_2 outperformed those based on baseline and prior_1 with good margins in terms of the Dice coefficient, and lesion-wise precision and recall.

**Table 3:** Results from 3D tumor segmentation using 3D UNet model across different cancer types. Dice, HD95, and ASD are voxel-level metrics, while Precision and Recall are lesion-wise metrics. Statistical tests were conducted using Wilcoxon-signed rank test between the baseline and prior_2 models as well as between the prior_1 and prior_2 models. Here, “*” indicates statistical significance, and “ns” indicates non-significance.

Method	Disease	**Table:** Dice (*μ* ± *σ*)	**Table:** HD95 (*μ* ± *σ*)	**Table:** ASD (*μ* ± *σ*)	**Table:** Precision (*μ* ± *σ*)	**Table:** Recall (*μ* ± *σ*)
3D UNet (baseline)	Lymphoma	0.70 ± 0.24 (*)	25.71 ± 43.24	6.56 ± 19.62	0.78 ± 0.31	0.74 ± 0.28
	Lung Cancer	0.76 ± 0.15 (*)	33.51 ± 40.02	7.52 ± 11.31	0.83 ± 0.23	0.62 ± 0.25
	Melanoma	0.59 ± 0.28 (*)	52.54 ± 66.36	8.54 ± 17.71	0.79 ± 0.31	0.75 ± 0.31

3D UNet (prior_1)	Lymphoma	0.71 ± 0.25 (*)	31.14 ± 54.84	8.32 ± 34.43	0.81 ± 0.25	0.77 ± 0.25
	Lung Cancer	0.76 ± 0.17 (ns)	33.38 ± 40.96	7.39 ± 15.48	0.81 ± 0.24	0.71 ± 0.24
	Melanoma	0.64 ± 0.28 (*)	50.58 ± 67.82	11.59 ± 30.40	0.80 ± 0.26	0.79 ± 0.28

3D UNet (prior_2)	Lymphoma	0.74 ± 0.22	23.58 ± 50.96	6.50 ± 35.02	0.83 ± 0.25	0.84 ± 0.23
	Lung Cancer	0.78 ± 0.15	28.63 ± 41.76	7.23 ± 16.34	0.87 ± 0.20	0.74 ± 0.24
	Melanoma	0.70 ± 0.24	43.83 ± 68.44	6.44 ± 17.25	0.82 ± 0.24	0.86 ± 0.22

**Table 4:** Results from 3D tumor segmentation using dynUNet model across different cancer types. Dice, HD95, and ASD are voxel-level metrics, while Precision and Recall are lesion-wise metrics. Statistical tests were conducted using Wilcoxon-signed rank test between the baseline and prior_2 models as well as between the prior_1 and prior_2 models. Here, “*” indicates statistical significance, and “ns” indicates non-significance.

Method	Disease	**Table:** Dice (*μ* ± *σ*)	**Table:** HD95 (*μ* ± *σ*)	**Table:** ASD (*μ* ± *σ*)	**Table:** Precision (*μ* ± *σ*)	**Table:** Recall (*μ* ± *σ*)
dynUNet (baseline)	Lymphoma	0.70 ± 0.23 (*)	33.96 ± 58.36	6.59 ± 28.56	0.74 ± 0.29	0.80 ± 0.25
	Lung Cancer	0.76 ± 0.16 (*)	40.45 ± 51.16	5.20 ± 6.60	0.74 ± 0.26	0.71 ± 0.22
	Melanoma	0.62 ± 0.28 (*)	60.68 ± 70.61	7.55 ± 23.22	0.73 ± 0.31	0.81 ± 0.27

dynUNet (prior_1)	Lymphoma	0.70 ± 0.23 (*)	35.92 ± 84.56	7.25 ± 23.84	0.75 ± 0.28	0.80 ± 0.29
	Lung Cancer	0.77 ± 0.16 (*)	35.76 ± 42.39	5.71 ± 13.25	0.76 ± 0.25	0.75 ± 0.23
	Melanoma	0.67 ± 0.28 (*)	51.17 ± 74.16	5.05 ± 15.72	0.78 ± 0.27	0.85 ± 0.24

dynUNet (prior_2)	Lymphoma	0.75 ± 0.22	22.95 ± 49.81	7.32 ± 35.17	0.84 ± 0.24	0.82 ± 0.24
	Lung Cancer	0.80 ± 0.14	31.56 ± 41.95	5.47 ± 13.26	0.81 ± 0.24	0.76 ± 0.23
	Melanoma	0.70 ± 0.23	42.07 ± 63.80	6.46 ± 18.19	0.82 ± 0.26	0.86 ± 0.23

**Table 5:** Results from 3D tumor segmentation using nnUNet model across different cancer types. Dice, HD95, and ASD are voxel-level metrics, while Precision and Recall are lesion-wise metrics. Statistical tests were conducted using Wilcoxon-signed rank test between the baseline and prior_2 models as well as between the prior_1 and prior_2 models. Here, “*” indicates statistical significance, and “ns” indicates non-significance.

Method	Disease	**Table:** Dice (*μ* ± *σ*)	**Table:** HD95 (*μ* ± *σ*)	**Table:** ASD (*μ* ± *σ*)	**Table:** Precision (*μ* ± *σ*)	**Table:** Recall (*μ* ± *σ*)
nnUNet (baseline)	Lymphoma	0.74 ± 0.23 (*)	25.65 ± 44.53	6.32 ± 30.48	0.77 ± 0.26	0.85 ± 0.23
	Lung Cancer	0.80 ± 0.13 (ns)	27.23 ± 44.53	5.89 ± 12.34	0.77 ± 0.22	0.83 ± 0.18
	Melanoma	0.65 ± 0.27 (*)	48.04 ± 65.32	6.87 ± 19.43	0.71 ± 0.27	0.87 ± 0.22

nnUNet (prior_1)	Lymphoma	0.73 ± 0.25 (*)	25.88 ± 45.68	6.76 ± 32.53	0.76 ± 0.24	0.84 ± 0.23
	Lung Cancer	0.80 ± 0.14 (ns)	26.15 ± 42.93	5.19 ± 13.63	0.76 ± 0.23	0.83 ± 0.20
	Melanoma	0.65 ± 0.28 (*)	50.15 ± 67.62	6.54 ± 18.43	0.67 ± 0.28	0.86 ± 0.24

nnUNet (prior_2)	Lymphoma	0.76 ± 0.23	22.58 ± 46.54	6.89 ± 34.76	0.81 ± 0.23	0.86 ± 0.22
	Lung Cancer	0.80 ± 0.15	27.63 ± 43.54	5.14 ± 13.57	0.77 ± 0.23	0.83 ± 0.21
	Melanoma	0.68 ± 0.24	44.69 ± 67.85	6.32 ± 17.53	0.73 ± 0.26	0.87 ± 0.22

Table 6 presents the performance of the segmentation networks (3D UNet, dynUNet, nnUNet) on an internal test set from the U-CAN cohort using different methodologies (baseline, prior_1, prior_2). The nnUNet (prior_2) method demonstrated the best performance among all the models evaluated.

**Table 6:** Results of 3D tumor segmentation on the internal test set from the U-CAN dataset using different networks and input data. Since U-CAN cohort contained only DLBCL cases, networks (3D UNet, dynUNet, nnUNet) trained on lymphoma cases only were used for testing purposes.

Model	Inputs				Dice
	CT	SUV	Prior_1	Prior_2	
	✓	✓			0.4491
3D UNet (lymphoma)	✓	✓	✓		0.4652
	✓	✓		✓	0.5165

	✓	✓			0.5042
dynUNet (lymphoma)	✓	✓	✓		0.5132
	✓	✓		✓	0.5367

	✓	✓			0.5483
nnUNet (lymphoma)	✓	✓	✓		0.5368
	✓	✓		✓	0.5632

**Figure 2:**
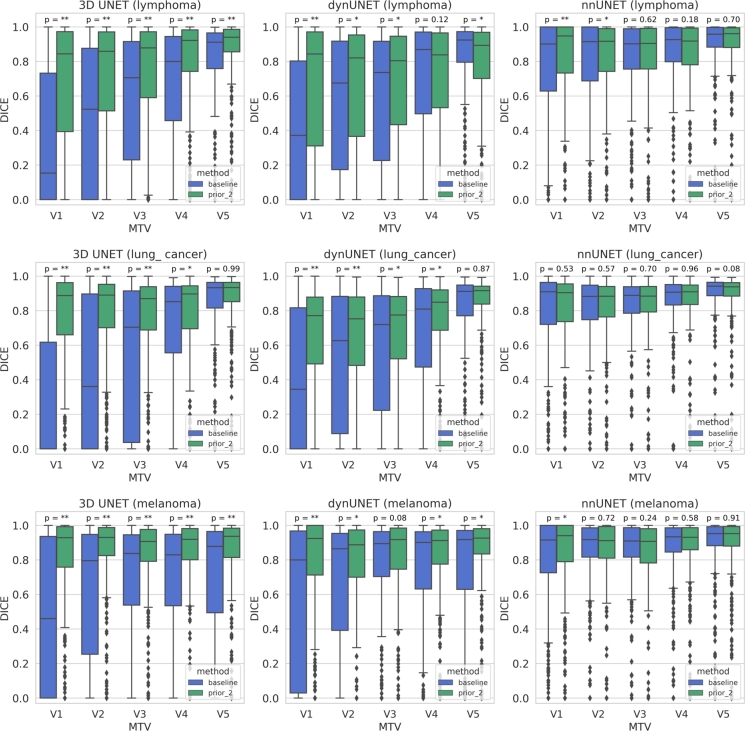
Lesion-wise comparison of tumor segmentation accuracy (Dice) between baseline and prior_2 methods for the three networks (3D UNet, dynUNet, and nnUNet) across different metabolic tumor volume (MTV) groups, categorized as V1, V2, V3, V4, and V5. For lymphoma: V1 (≤ 1 ml), V2 (1-2 ml), V3 (2-4 ml), V4 (4-14 ml), V5 (≥ 14 ml); for lung cancer: V1 (≤ 2 ml), V2 (2-3.5 ml), V3 (3.5-7.3 ml), V4 (7.3-23 ml), V5 (≥ 23 ml); for melanoma V1 (≤ 1 ml), V2 (1-1.5 ml), V3 (1.5-3 ml), V4 (3-8 ml), V5 (≥ 8 ml). Statistical comparison of the Dice coefficient between the baseline and prior_2 methods, using the Wilcoxon signed rank test, is also shown across different MTV groups. Here, “*” corresponds to p-value within range 0.001-0.05 and “**” corresponds to p-value less than 0.001.

**Figure A.1:**
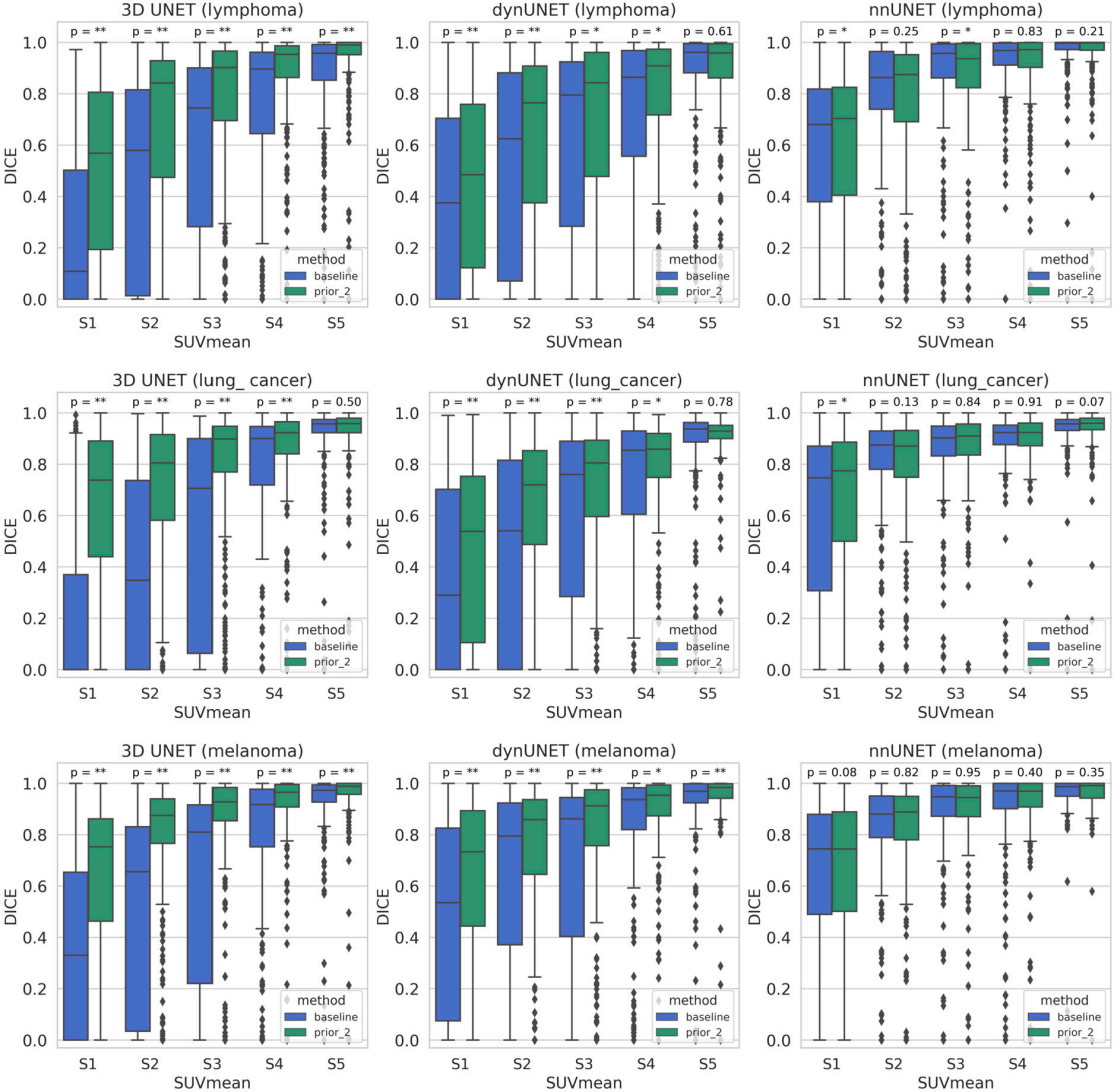
Lesion-wise comparison of tumor segmentation accuracy (Dice) between baseline and prior_2 methods for the three networks (3D UNet, dynUNet, and nnUNet) across different SUVmean groups, categorized as S1, S2, S3, S4, and S5. For lymphoma: S1 (1.95-3.84), S2 (3.84-4.52), S3 (4.52-5.25), S4 (5.25-6.33), S5 (6.33-18.42); for lung cancer: S1 (1.18-2.38), S2 (2.38-2.72), S3 (2.72-3.15), S4 (3.15-4.04), S5 (4.04-13.86); for melanoma S1 (1.82-3.96), S2 (3.97-4.81), S3 (4.81-5.67), S4 (5.67-7.10), S5 (7.12-31.06). Statistical comparison of the Dice coefficient between the baseline and prior_2 methods, using the Wilcoxon signed rank test, is also shown across different MTV groups. Here, “*” corresponds to p-value between 0.001-0.05 and “**” corresponds to p-value less than 0.001.

Fig. 2 illustrates the comparison of tumor segmentation accuracy (Dice) between the baseline and prior_2 methods, using 3D UNET, dynUNET, and nnUNet across different metabolic tumor volume (MTV) groups, among all three cancer types. Models based on prior_2 enhance the tumor segmentation performance as compared to those based on baseline, across most of the MTV groups. A similar comparison between baseline and prior_2 models across different SUVmean groups is provided in Fig. A.1 of the appendix section.

Tables 7 [a]-[f] display the confusion matrices illustrating the comparison between the “baseline” and “prior_2” methods as well as between the “prior_1” and “prior_2” methods using a 3D UNet model, focusing on the total FN lesion count. The results indicate that the “prior_2” method identifies a larger number of lesions across all cancer types, some of which are missed by the “baseline” or “prior_1” method. On the contrary, the number of lesions detected by the “baseline” or “prior_1” method but missed by the “prior_2” method is relatively small. Similar comparisons using dynUNet and nnUNet models are shown in Table A.1, Table A.2 of the appendix section.

**Table 7:**
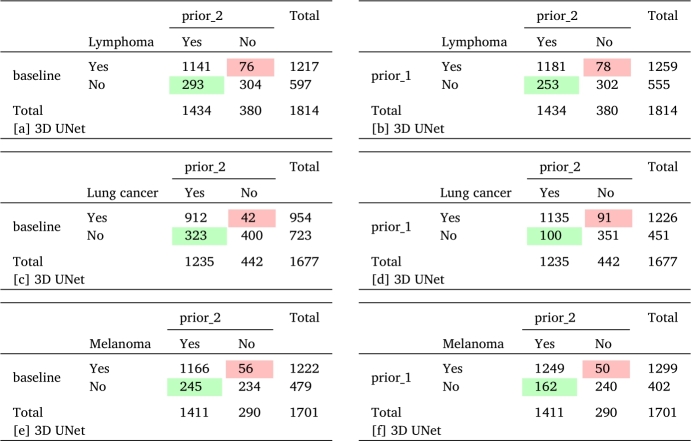
Comparison of the total number of individual false negative lesions between the “baseline” and “prior_2” methods as well as between the “prior_1” and “prior_2” methods using 3D UNet across different cancer types. Here, “Yes” represents the number of detected lesions and “No” represents the number of undetected lesions. In the case of melanoma, one patient was excluded from calculations due to the presence of exceptionally large number of lesions.

Table 8 gives a brief overview of the tumor segmentation results in whole-body PET/CT from several methods that participated in the autoPET grand challenge 2022.

**Table 8:** Overview of the 5-fold cross validation (CV) results for whole-body PET/CT tumor segmentation from the autoPET Grand Challenge 2022, as reported by other researchers.

Method	Model	Description	Dice	Comments
1 [20]	UNet	2D UNet based tumor segmentation with 5-fold CV strategy	0.69	Outperformed by our method.

2 [21]	UNet	Network takes PET and CT as input and outputs 8 channels, one of which is the true segmentation mask and others are auxiliary channels. 40 images were set aside for validation.	0.80	CV results are not available. Results reported on a set aside test set.

3 [22]	nnUNet + Swin UNetR	A 5-fold cross-validation was employed with stratification based on sex and diagnosis, and late fusion was applied to enhance the overall Dice.	0.72	Outperformed by our method.

4 [24]	nnUNet	Introduced a false positive reduction network for enhanced segmentation performance.	0.93	CV results are not available. Only results from preliminary test set reported.

5 [25]	UNet	Simple UNet based training and validation was done with an input size of (192, 192, 192). 103 images were set aside for validation.	0.75	CV results are not available. Results reported on a set aside validation set.

6 [26]	nnUNet	Proposed a joint (2D-3D models) whole-body lesion segmentation approach with a patch size of (128, 128, 128).	0.79	Performed 5-fold CV but reported results solely on the best performing fold 1 and 2.

7 [27]	nnUNet	Proposed a 2 step approach: first, generating a prior using the normal appearance autoencoder, and second, incorporating this prior into the segmentation network.	0.70	Outperformed by our method.

8 [28]	nnUNet	Proposed to use nnUNet with Graph convolutional network (GCN) refinement. 30 images were set aside for validation.	0.76	CV results are not available. Results reported on a set aside validation set.

9 [18]	3D UNet	Proposed to use a segmentation prior- based approach for enhanced tumor segmentation.	0.70	Outperformed by our method.

Ours	nnUNet	Described in section 3 of this paper.	0.74	-

**Figure 3:**
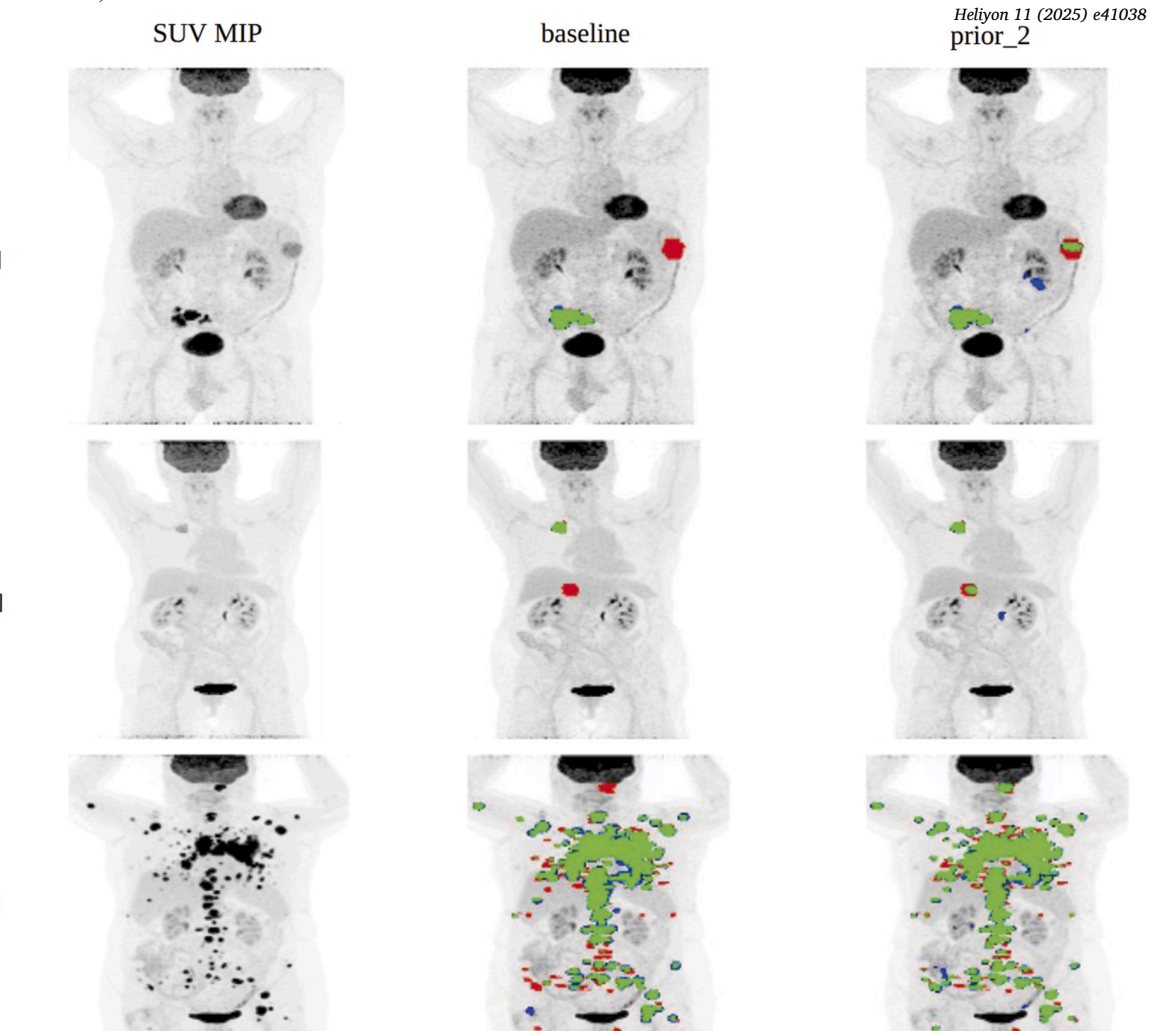
Visualization of tumor prediction results that are missed by the nnUNet (baseline) model but are picked up by the nnUNet (prior_2) model. TPs (True positives) are shown in green, FNs (false negatives) in red, FPs (false positives) in blue. Figures shown in [a], [b], [c] are examples of tumors that are difficult to segment, from the autoPET cohort, because of their small size or low FDG uptake.

Fig. 3 [a] - [c] shows the visualization of patients with tumors difficult to segment (especially small and low FDG-uptake ones) that are missed by the nnUNet (baseline) model but are detected by the nnUNet (prior_2) model.

Detailed comparison between 3D UNet, dynUNet, and nnUNet is provided in Table A.3 of the appendix section to assess their complexity and computational requirements.

A detailed comparison between the baseline, prior_1, and prior_2 methods, in terms of computational costs and other technical parameters, is provided in Table A.4 of the appendix section.

Finally, we tried to find the optimal patch size for the 3D tumor segmentation task by training a 3D UNet using various patch sizes. We found that the patch size of (160, 160, 160) gave the best results compared to other patch sizes, as shown in Table A.5 in the appendix section.

## 4 Discussion

In this study, we have introduced a tissue-wise multi-channel projection-based approach to reconstruct a segmentation prior dedicated for automated tumor segmentation in whole-body FDG-PET/CT. In our proposed method, the segmentation prior was used as an additional input channel to enhance the overall segmentation performance. We have demonstrated the effectiveness of our proposed approach (prior_2) in significantly improving the tumor segmentation performance across various cancer types in the autoPET cohort, compared to a baseline and a previously published prior_1 [18] method. See Table 3, Table 4, Table 5 for details. In addition, the prior_2 method showed equal or superior performance compared to the baseline and prior_1 method for all three networks evaluated, with nnUNet as the best performing network overall. However, the cross-validation using nnUNet was observed to be computationally expensive, requiring higher RAM capacity compared to 3D UNet and dynUNet (see Table A.3 of the appendix section).

In an internal test set (U-CAN cohort with DLBCL cases), the prior_2 method outperformed the baseline and prior_1 methods in terms of Dice coefficient across different segmentation networks (3D UNet, dynUNet, nnUNet), as shown in Table 6. This signifies that the prior_2 method has superior generalization performance on previously unseen dataset, and that inclusion of the additional information provided by the prior_2 method enhances the segmentation performance compared to the baseline and prior_1 methods.

Our approach involves segmentation of tumors from multi-channel multi-angled SUV projections to create the segmentation priors, with the CT information used to generate the multi-channel SUV projections. A key advantage of this approach lies in the simplicity of training a 2D segmentation network to estimate the approximate location of lesions, compared to the more complex task of 3D segmentation (without the segmentation prior). This is especially helpful for extremely small lesions that otherwise are difficult to segment, as small lesions are accentuated relative to normal tissue in MIPs due to the projection process. As a result, there is a notable reduction in class imbalance between tumor and non-tumor pixels, as well as less ambiguity at tumor boundaries. This characteristic makes the 2D segmentation network more adept at accurately distinguishing tumors from background. Moreover, since we are explicitly segmenting the same lesions from multiple angles, the network's tendency to overlook a lesion from one angle is compensated by its detection capability from other angles. The ultimate goal is to segment the maximum number of lesions from multiple directions. When applying backprojection, regions with higher overlapping tumor regions between projections receive higher weighting compared to less overlapping tumor regions. By using the segmentation prior, the 3D segmentation network can capture extremely small and difficult tumor regions more effectively.

In the context of 2D tumor segmentation, the $SUV_{org}^{MIP}$ channel is potentially the primary source of tumor-related contrast. However, our hypothesis suggested that inclusion of multi-channel projections, such as bone, lean tissue, adipose tissue, and air, alongside $SUV_{org}^{MIP}$, could significantly improve the 2D tumor segmentation. The rationale behind this hypothesis originates from the observation that the inclusion of additional channels could offer supplementary tumor-related information, thereby complementing the data provided by $SUV_{org}^{MIP}$ channel. Also, it is important to acknowledge that not all information from the 3D SUV image can be preserved within a $SUV_{org}^{MIP}$ channel due to the projection process. Therefore, by extracting supplementary information in the form of $SUV_{bone}^{MIP}$, $SUV_{lean}^{MIP}$, $SUV_{adipose}^{MIP}$, and $SUV_{air}^{MIP}$, additional tumor related information, unavailable in the $SUV_{org}^{MIP}$, could be provided. This additional information enables the 2D network to effectively segment the challenging lesions. Consequently, the utilization of multi-channel inputs improves the overall 2D segmentation accuracy by complementing the information obtained from the $SUV_{org}^{MIP}$ and capturing tumor-related features that may be obscured in the $SUV_{org}^{MIP}$ alone. The results in Table 2 clearly demonstrate the improved performance achieved by employing multi-channel inputs compared to using the $SUV_{org}^{MIP}$ channel alone, across all cancer types. As a result, the integration of multi-channel MIPs leads to a more robust reconstruction of the segmentation prior, thereby enhancing the performance of 3D tumor segmentation. Table 3, Table 4, Table 5 demonstrates the superiority of the “prior_2” method (multi-channel input) over “prior_1” ($SUV_{org}^{MIP}$ alone), across all cancer types. While the prior_2 proved to be beneficial, particularly for 3D UNet and dynUNet, the advantages were less pronounced for nnUNet.

While Table 3, Table 4, Table 5 highlight the increased Dice coefficients achieved by the proposed method, the clinical relevance needs to be emphasized. The improved segmentation accuracy could directly be beneficial for automated quantification of tumor burden, a known prognostic factor for assessing disease progression and an appealing biomarker for clinical trials. In a more long-term perspective, accurate identification and quantification of individual tumors could improve and streamline cancer diagnostics for positive effects on patient management and outcome.

In general, the 3D baseline method has limited performance in detecting very small and low FDG uptake lesions due to higher class imbalance and low contrast. By incorporating a segmentation prior, the network can learn to focus on highlighted regions of probable tumors, resulting in more reliable segmentation masks. This approach proves particularly effective in detecting small and low FDG uptake lesions, as demonstrated in Fig. 2 and Fig. A.1 of the appendix section. Fig. 2 demonstrates significant improvement in tumor segmentation Dice across various MTV groups, particularly for lesions with low MTV (such as V1), in all cancer types. Similarly, Fig. A.1 in the appendix section demonstrates significant improvement in tumor segmentation Dice across various SUVmean groups (here SUVmean corresponds to the mean SUV of the individual lesions), especially for lesions with lower SUVmean (S1), in all cancer types. This is important in a clinical setting, where the detection of challenging tumors, especially small tumors and tumors with low FDG uptake is crucial as they are more likely to be overlooked by radiologists during manual evaluation. Neglecting these tumors could lead to their spread to healthy tissues, undermining the effectiveness of treatment.

Table 7 provides a quantitative overview of the number of lesions detected by the prior_2 method but missed by the baseline or prior_1 methods, and vice-versa, across all cancer types using the 3D UNet model. The majority of FN lesions escaping detection by the baseline method were successfully detected by the prior_2 method (see Table 7, [a] Lymphoma = 293; [c] Lung cancer = 323; [e] Melanoma = 245). However, some of the FN lesions missed by the prior_2 method were captured by the baseline method (see Table 7, [a] Lymphoma = 76; [c] Lung cancer = 42; [e] Melanoma = 56). This discrepancy can be attributed to uncertainties in the overall optimization process. Nevertheless, in general, the prior_2 method demonstrated superior performance compared to the baseline method and was able to segment a greater number of individual lesions without any additional FPs (see Table 3, Table 4, Table 5). Furthermore, there were few instances where both methods struggled to accurately segment the target lesions (see Table 7, [a] Lymphoma = 304; [c] Lung cancer = 400; [e] Melanoma = 234). A similar comparison between the prior_2 and prior_1 methods are shown in Table 7 [b], [d], [f]. Additionally, quantitative analysis of the number of detected lesions using dynUNet and nnUNet models is provided in Tables A.1 [a]-[f] and A.2 [a]-[f] in the appendix section. In general, prior_2 demonstrated superior performance in terms of additional lesions detected compared to prior_1 and the baseline models, both for 3D UNet and dynUNet. However, the improvement was smaller in the case of nnUNet.

During a follow-up analysis, we found that certain FP predictions by the prior_2 method corresponded to actual lesions previously missed by the human annotator (FP predictions determined by a radiologist with 5 years of experience). Manual detection of these additional FN lesions would require a significant investment of time and labor for re-assessment of the entire cohort. However, the proposed framework can provide valuable assistance to radiologists in expediting the comprehensive manual evaluation process, thereby increasing the likelihood of avoiding any lesion oversight. The segmentation prior can also inform about the tumor-related importance of different body regions as it resembles a probability distribution for tumors. It can be used by radiologists during cancer screening or follow-up analysis and can be adjusted manually, saving valuable time. It also directs the deep learning network's focus towards highlighted regions, reducing overall uncertainty in the optimization and leading to improved segmentation masks.

The autoPET grand challenge [29] has led to a surge in the development of automated whole-body PET/CT tumor segmentation methods. A comprehensive summary of tumor segmentation results from state-of-the-art methods from the grand challenge is summarized in Table 8 [20] [21] [22] [24] [25] [26] [27] [28]. Overall, our proposed method demonstrated superior performance compared to most methods. However, it is worth noting that a few methods cannot be directly compared to ours due to the absence of 5-fold cross-validation results.

While our proposed method demonstrated superior performance compared to the baseline method, it is important to consider the computational cost and training time for practical implementation in clinical settings. The proposed method requires higher computational resources compared to the baseline, particularly in terms of RAM capacity, during the reconstruction of the segmentation prior (see Table A.4 in the appendix section). However, it is noteworthy that while the proposed method delivers improved segmentation results, it does not increase the model complexity. As a result, the training times remain comparable to those of the baseline method.

Integrating the proposed tumor segmentation framework into clinical workflows requires a diverse dataset for robust model training and validation. It also presents several challenges, such as ensuring compatibility with existing systems like PACS and maintaining data privacy and security. Successful integration into clinical practice necessitates collaboration with healthcare professionals to gain clinical validation and acceptance through rigorous trials. Additionally, obtaining regulatory approval is essential to ensure the framework's safety, efficacy, and compliance with healthcare standards. Overcoming these challenges requires an iterative development process, ongoing validation to meet clinical standards, and ensuring that the framework is scalable and generalizable across various patient populations and healthcare facilities.

In future work, we intend to explore the use of iterative reconstruction with backprojection techniques to enhance the quality of the segmentation prior and investigate their impact on the overall tumor segmentation. We also intend to investigate the application of different smoothing based transfer functions, like sigmoid functions, as an alternative to hard thresholding on the CT HUs. Future plans also include application of the tissue-wise multi-channel PET/CT projections to predict clinical outcomes such as overall survival.

## 5 Limitations

In rare instances, our 2D segmentation network failed completely to segment the tumors using the multi-directional 2D projections. During such occurrences, the segmentation prior did not provide any valuable additional information. Consequently, integrating such priors into the 3D segmentation network does not yield any improvement compared to the baseline network. In fact, the Dice coefficient remains identical for baseline and prior networks in such cases.

## 6 Conclusion

We have introduced a multi-channel, multi-angled projection-based approach for the reconstruction of a segmentation prior for tumors in FDG-PET/CT images. Inclusion of the segmentation prior enhanced 3D tumor segmentation accuracy, outperforming the baseline across three cancer types, particularly improving detection of small and low-FDG uptake lesions often missed by radiologists. This highlights the potential of the proposed framework as a valuable tool for the radiologist to perform automated quantification of tumor volume with future potential to streamline lesion-wise monitoring and enable faster and more reliable follow-up evaluations.

## 7 Abbreviations

**Table:** 

PET	Positron Emission Tomography
CT	Computed Tomography
FDG	18F-fluorodeoxyglucose

**Table:** 

SUV	Standardized uptake value
HU	Hounsfield Units
WHO	World health organization
CNN	Convolutional neural network
MIP	Maximum intensity projection
TCIA	The Cancer Imaging Archive
DL	Dice loss
FL	Focal loss
HD95	Hausdorff distance
ASD	Average surface distance
TMTV	Total metabolic tumor volume
TP	True positive
FN	False negative
FP	False positive

## Ethics approval and consent to participate

Ethical approval to conduct retrospective image analysis on the autoPET and U-CAN datasets was obtained from the Swedish Ethical Review Authority with reference number Dnr 2023-02312-02. The study was conducted in accordance with relevant guidelines and regulations, including the Declaration of Helsinki.

## Consent for publication

Not Applicable.

## Funding

This study was supported by the Swedish Cancer Society (201303 PjF 01 H), Lions Cancer Fund Uppsala and Makarna Eriksson foundation.

## CRediT authorship contribution statement

**Sambit Tarai:** Writing – original draft, Visualization, Validation, Methodology, Investigation, Formal analysis, Conceptualization. **Elin Lundström:** Writing – review & editing, Validation, Supervision, Project administration, Investigation, Formal analysis. **Nouman Ahmad:** Writing – review & editing, Visualization, Formal analysis. **Robin Strand:** Writing – review & editing, Supervision, Formal analysis. **Håkan Ahlström:** Writing – review & editing, Validation, Supervision, Funding acquisition, Data curation. **Joel Kullberg:** Writing – review & editing, Validation, Supervision, Methodology, Investigation, Funding acquisition, Formal analysis.

## Declaration of Competing Interest

The authors declare the following financial interests/personal relationships which may be considered as potential competing interests:

Hakan Ahlstrom reports financial support was provided by 10.13039/501100002794Swedish Cancer Society. Joel Kullberg reports a relationship with Antaros Medical AB that includes: employment and equity or stocks. Hakan Ahlstrom reports a relationship with Antaros Medical AB that includes: employment and equity or stocks. Sambit Tarai reports a relationship with Antaros Medical AB that includes: employment. Sambit Tarai, Håkan Ahlström, Joel Kullberg report relationship with Antaros Medical AB, which include employment and equity or stocks. Elin Lundström, Nouman Ahmad, and Robin Strand report no competing interests. If there are other authors, they declare that they have no known competing financial interests or personal relationships that could have appeared to influence the work reported in this paper.

## Figures and Tables

**Table A.1:**
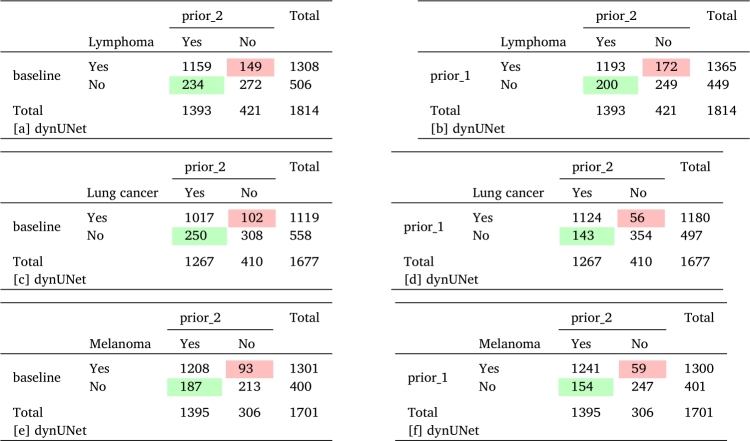
Comparison of total number of individual false negative lesions between the “baseline” and “prior_2” methods as well as between the “prior_1” and “prior_2” methods using dynUNet across different cancer types. Here, “Yes” represents the number of detected lesions and “No” represents the number of undetected lesions. In case of melanoma, one patient scan was excluded from calculations due to the presence of exceptionally large number of lesions.

**Table A.2:**
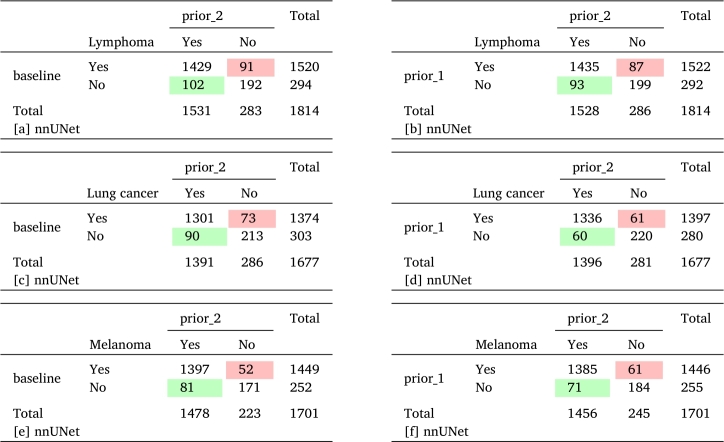
Comparison of total number of individual false negative lesions between the “baseline” and “prior_2” methods as well as between the “prior_1” and “prior_2” methods using nnUNet across different cancer types. Here, “Yes” represents the number of detected lesions and “No” represents the number of undetected lesions. In case of melanoma, one patient scan was excluded from calculations due to the presence of exceptionally large number of lesions.

**Table A.3:** Comparison of different segmentation networks (3D UNet, dynUNet, nnUNet) across various criteria. In all cases, UNet was used as the backbone architecture.

Comparison criteria	Segmentation networks		
	3D UNet	dynUNet	nnUNet
[1] Total trainable parameters	4807345	62839746	49339250
[2] Model size (MB)	57.9	377.2	250
[3] Training time per epoch (seconds)	210	220	260
[4] Validation time per epoch (seconds)	60	80	70
[5] Inference time per subject (seconds)	4	6	60
[6] Maximum batch size with current hardware	4	1	1
[7] Internal RAM usage	low	medium	high

**Table A.4:** Comparison of different segmentation approaches (baseline, prior_1, prior_2) across various criteria. In all cases, 3D UNet was used as the backbone architecture.

Comparison criteria	Segmentation approach		
	baseline	prior_1	prior_2
[1] Total trainable parameters	4807345	4808209	4808209
[2] Model size (MB)	57.9	58.2	58.2
[3] Training time per epoch (seconds)	210	210	210
[4] Validation time per epoch (seconds)	60	60	60
[5] Segmentation prior	No	Yes	Yes
[6] Segmentation prior generation (RAM usage)	No	high	high
[7] Performance	Good	Better	Best

**Table A.5:** Comparison of segmentation accuracy using different input patch sizes with the 3D UNet as the architecture. Data from all cancer types (lymphoma, lung cancer, melanoma) from the autoPET cohort were consolidated, with 80% used for training and the remaining 20% for validation. The Dice coefficient is reported for the validation subset comprising 20% of the data.

Inputs			Patch size	Network	Dice
CT	SUV	prior_2			
✓	✓	✓	(96, 96, 96)	3D UNet	0.6213
✓	✓	✓	(128, 128, 128)	3D UNet	0.6285
✓	✓	✓	(160, 160, 160)	3D UNet	0.6618
✓	✓	✓	(208, 208, 208)	3D UNet	0.6427

## Data Availability

The autoPET dataset is publicly available at The Cancer Imaging Archive (TCIA). The U-CAN dataset is not publicly available but could be made available subject to request and approval. The code for the automated framework is available in the following Github repository: https://github.com/sambittarai/Tumor-segmentation-from-PET-CT-followed-by-clinical-parameter-estimation.
